# Early Intervention in Families with Preterm Infants: A Review of Findings from a Randomized Controlled Trial Following Children Up to 9 Years of Age

**DOI:** 10.3390/children9040474

**Published:** 2022-03-30

**Authors:** Stein Erik Ulvund

**Affiliations:** Department of Education, University of Oslo, 0317 Oslo, Norway; s.e.ulvund@iped.uio.no

**Keywords:** prematurity, early intervention, longitudinal study, parent-child interaction, cognitive development, social development, behavioral problems, parenting stress

## Abstract

The Tromsø Intervention Study on Preterms (TISP) randomized 146 preterm born children (<2000 g) either to the Mother-Infant Transaction Program-Modified (MITP-M, *n* = 72) or to a preterm control group (*n* = 74). In addition, 75 full-term babies were followed up until 9 years of age. TISP was conducted at the University Hospital Northern Norway (UNN) and only infants who did not have congenital anomalies and families where the mothers’ native language was Norwegian were included. The study investigates the effect of MITP-M on cognitive and social development including behavioral problems, quality of life and stress in the family. The results have so far been published in various journals. The aim of this article is to give a comprehensive overall presentation of the main findings and discuss implications for clinical practice and further research. Parents in the intervention group were superior in “reading” their infants’ temperament, and at 3, 5 and 7 years of age the intervention group scored significantly higher on well-known tests of cognitive outcome. At 9 years of age, the intervention group had fewer attentional problems, better school achievements and a better quality of life. From the first year onwards, mothers and fathers in the intervention group reported lower levels of stress than parents of in the preterm control group.

## 1. Introduction

Preterm birth is defined as birth at less than 37 completed weeks of gestation, low birth weight (<2500 g) or very low birth weight (<1000 g). It is well known that preterm infants are at risk of delayed cognitive and social development, learning disabilities, increased occurrence of behavioral problems and the families often suffer from stress. The lower gestation age and birth weight are, the higher the risk of complications. Various early intervention strategies have been attempted to improve long-term outcomes [1]. The results are unclear and many of the studies have followed up relatively small samples. Variability in definitions and measurement as well as quality of the individual studies are among the methodological factors that can explain inconsistencies in the results. Some of the most cited intervention studies are based on the Mother-Infant Transaction Program (MITP), which was developed for and used in the Vermont Intervention Study [2,3]. In a meta-review on the effectiveness of various early intervention programs for parents and preterm infants, the MITP was the second most frequently reported intervention followed by Modified-Mother Infant Transaction Program [1]. The original Vermont study was based on the transactional model of development [4]. The study was small and the results showed improved cognitive functioning in the intervention group at 7 and 9 years of age. When we started to plan our study, the results from the Vermont study seemed promising. Therefore, we wanted to assess the intervention procedure on a larger sample. 

In March 1999, the Tromsø Intervention Study on Preterms (TISP) was initiated and conducted at the University Hospital Northern Norway (UNN) located in the city of Tromsø. UNN is serving the two northernmost counties in the country with about 230,000 inhabitants. TISP used a modified version of the MITP (MITP-M) and is based on the transactional model of development. This model places equal emphasis on child and parents and disturbed infant-child-interactions are assumed to have a major influence on the child’s cognitive and social development. The mother’s conception of and response to the infant is largely influenced by the infant’s temperament, which again influences the infant’s experience with the parents. These mutual influences are assumed to be cornerstones in infants’ development. Further, the intervention tries to sensitize parents to cues of their infants, especially signs of stimulus overload (e.g., head turning), distress (e.g., crying) and readiness for interaction. An important point in MITP-M is also to enable parents to appreciate the unique characteristics and developmental potential of their infants.

The results of the TISP up to 9 years of age are now completed and have been published in several different international journals. Despite their significant contribution to the field and high citation rates, it has not been easy to keep track of the overall results in the articles. The main aim of this article is to give an overall relatively short and comprehensible presentation of the main findings of the study, and discuss implications for clinical practice and further research. 

## 2. Material and Methods

### 2.1. Participants

**Figure 1:**
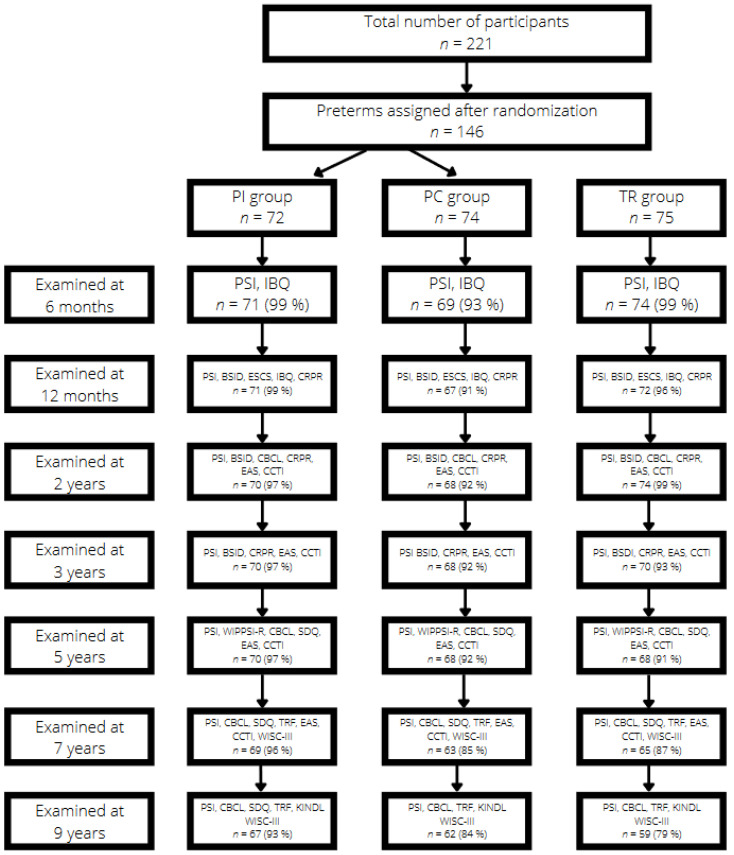
Study flow diagram from ages 6 months to 9 years. BSID = Bayley Scales of Infant Development; CBCL = Child Behavior Checklist; CCTI = Colorado Childhood Temperament Inventory questionnaire; CRPR = Child Rearing Practice Report; EAS = Emotionality, Activity and Sociability questionnaire; ESCS = Early Social Communication Scales; IBQ = Infant Behavior Questionnaire; PSI = Parenting Stress Index: KINDL = Questionnaire for measuring quality of life for children and adolescents; SDQ = Strengths and Difficulties Questionnaire; TRF = Teacher Report Form; WIPPSI-R = Wechsler Preschool Primary Scale of Intelligence; WISC- III = Wechsler Primary Intelligence Scale for Children.

The period of recruitment spanned from March 1999 to August 2002. All infants with birth weights below 2000 g who did not have congenital anomalies were recruited, except families where the mothers’ native language was not Norwegian. Due to the character of the study, triplets were excluded. Infants who were not born at UNN were included if they were transported to the hospital within a week. Infants who were not expected to be able to follow our planned test procedures due to neurologic sequelae were excluded from the study. The coordinating nurse informed the families about the study two weeks before hospital discharge. Written informed consent was obtained if the parents agreed to participate. Randomization was arranged in random blocks of four and six using computer-generated random numbers and stratified gestation (<28 or ≥28 weeks). The infants were assigned into either the preterm intervention group (*n* = 72) or the preterm control group (*n* = 74) (see Figure 1). A group of term controls (*n* = 75) with gestational age ≥ 37 weeks and birth weight >2800 g was recruited from the well-infant nursery nearby the hospital. The regional committee for medical research and the Norwegian Data Inspectorate approved the study.

### 2.2. Intervention

Eight experienced neonatal nurses working at the hospital were specially trained for the intervention. As already mentioned, TISP used a modified version of the MITP (MITP-M). The modification included an initial debriefing session in which the parents could talk about experiences in connection with the hospital stay. The parents got the opportunity to express feelings such as grief, disappointment, possible reasons for the preterm birth and concerns for the future development of their infants. This session was also meant to encourage the parents to participate in the intervention sessions. After the initial session, eight trained neonatal nurses implemented 1-h daily sessions with parents and infants over 7 days. The sessions started 1 week before discharge. Each session focused on different aspects such as *reflexes*, *self-regulation*, *sign of distress* and *demonstrations* of how parents could bring the infant into a quiet alert state, ready for social interaction. It was also addressed how sensitive and responsive child-care could be a part of daily routines. The same intervention nurse followed up the daily sessions with home visits on the 3rd, 14th, 30th and 90th days after discharge. These four home visits focused on (a) experiences after discharge (b) adjustment to the home environment, (c) individual characteristics of the child (temperament), (d) how to guide and stimulate the infant, and (e) an evaluation of the intervention program. The nurses kept detailed logbooks of every interventional session, which were regularly reviewed by the coordinating nurse and a psychologist to ensure consistency of the intervention and thus procedural fidelity.

The preterm control group (*n* = 74) followed UNN’s standard protocol for discharge. This included an offer of training in infant massage, a clinical examination including visual and hearing screening and a consultation with one of the medical doctors. The term control group (*n* = 75) was given a clinical examination on the third day of life and no other interventions were offered. 

## 3. Instruments and Power Calculations

An overview of the instruments relevant for this article is presented in the flow diagram (see Figure 1). All self-report instruments were translated to Norwegian. The study size was calculated based on a difference between the preterm groups on developmental outcome measures used in the study. The power analysis showed that 60 preterm infants needed to be included in intervention and control groups. As it can be seen from the flow diagram, drop-outs over 9 years were small and no groups dropped below the limit of 60 subjects except for the term control group at 9 years of age (*n* = 59).

## 4. Outcomes

### 4.1. Cognitive Development

The outcomes of cognitive functioning at 2 years corrected age were assessed by the *Bayley Scales of Infant Development, Second edition* (BSID-II) [5]. When adjusting for education of the parents, there were no significant differences between the intervention and control groups at this age level [6]. BSID-II was also used at 3 years. At 5 years of age the *Wechsler Preschool Primary Scale of Intelligence–Revised* (WIPPSI-R) was used to measure the outcome of cognitive functioning [7]. At 3 and 5 years of age, there was a difference in favor of the intervention group and significantly more children in this group had IQ-scores ≥ 85. Also, at ages 3 and 5, adjusting for maternal education led to a somewhat reduced difference between the groups [8]. At 5 years of age, the difference in mean IQ-scores were within the clinically significant range (≥5 points). Significantly more children in the intervention group scored within a normal range (scores of ≥85). The main conclusion was that MITP-M improved cognitive outcome at 5 years corrected age.

At 7 and 9 years of age, the intervention group scored consistently higher on the *Wechsler Intelligence Scale for Children* (WISC-III) [9]. However, the only significant difference was found on the Verbal Comprehension Index at 7 years of age [10]. Although most of the differences in scores between the groups were not significant at 7 years of age, the scores were in the range of 4–5 points, which is a relatively large difference. Thus, these findings might be clinically relevant. At 9 years of age, the difference between the groups was only 1–2 IQ points. In another report [11], teachers who were unaware of the history of their pupils reported better academic performance in the intervention group than in the preterm control group at 9 years of age (*Teacher’s Report Form*, TRF) [12]. School achievements were at the statistically same level as term controls. 

### 4.2. Early Social Development and Parent-Child Interaction

As mentioned earlier infant temperament plays a crucial role in infant-parent interactions. Therefore, it was investigated to what extent the MITP-M influences parents’ perception of temperamental constructs (emotionality, activity level, sociability, shyness and sooth ability) from 2 to 7 years of age [13]. Temperament was reported by mothers and fathers separately on questions described in the *Emotionality, Activity and Sociability questionnaire* (EAS) [14] and in the *Colorado Childhood Temperament Inventory* (CCTI) [15]. The main finding was that parents in the intervention group reported lower levels of negative emotionality in their children (e.g., cries and gets upset easily) compared to the control group. For mothers the difference in reported negative emotionality was stable from 2 to 7 years. Fathers in the intervention group perceived their children as easier to soothe at all age levels. In conclusion, the MITP-M affected both mothers’ and fathers’ perception of their infants on several temperamental constructs. 

Prematurely born infants have shown deficiencies in various areas of social communication. At 12 months, effects of the TISP on social interaction measured by the *Early Social Communication Scales* (ESCS) [16] were studied with special focus on *joint attention* [17]. Joint attention consists of looking at objects, pictures, people or events together with an adult and has been shown to be of great importance in preterms’ early learning and development [18]. Infants in the preterm intervention group scored significantly higher than preterm controls on the dimensions Initiating Joint Attention, Initiating Object Requests and Responding to Social Interaction. Girls outperformed boys on all communication types measured [17]. Thus, an intervention implemented during the neonatal period can be advantageous for joint attention in preterm infants. 

However, another report from the same age level showed that preterm intervention infants with low *regulatory competence*, as measured by the *Infant Behavior Questionnaire* (IBQ) [19], had higher scores on Responding to Joint Attention than preterm control infants with low regulatory competence [20]. A sensitizing intervention implemented by the MITP-M may thus allow an alternative outlet for preterm infants low in regulatory competence. Regulatory competence covers behaviors that can assist parents to respond in a manner that allows organized responses to their preterm baby and supports their infants’ self-regulatory competence. In this context, it should be mentioned that mothers in the intervention group reported significantly more appropriate nurturing child-rearing practices than control-mothers at 12 and 24 months based on the *Child Rearing Practices Report* (CRPR) [21]. Thus, it was concluded that MITP-M might also lead to more nurturing child rearing among preterm mothers [22].

### 4.3. Behavioral Problems and Mental Health

At 2 years of age, behavioral problems were assessed by the *Child Behavior Checklist/2–3* (CBCL) [23]. The intervention group scored consistently lower on all CBCL scales, but the differences were not significant [6]. Probably, possible non-optimal parent-child interactions develop over some time before behavioral problems are more clearly revealed. This interpretation is supported by the findings at 5 years of age where behavioral problems were assessed by CBCL and the *Strengths and Difficulties Questionnaire* (SDQ) [24]. At this age level, parents in the intervention group reported significantly less behavioral problems on both instruments, and significantly more children in the preterm control group scored within the clinical area on both instruments [25]. It seems likely that the MITP-M facilitated development indirectly through more favorable parent-infant transaction patterns over time (“sleeper effect”).

At 7 and 9 years of age, parents (CBCL) and teachers (SDQ) reported that the intervention group had fewer attentional problems and better adaptation to school compared to preterm controls. At 9 years of age, teachers reported that the occurrence of difficulties in the intervention group was at the statistically same level as for term controls [11].

Quality of life (QoL) was also examined at age 9 where children and parents reported on the *Kinder Lebensqualität Fragbogen* [26,27] (KINDL for children, KINDL for parents). This was the first time that children had the opportunity to report outcomes independently of their parents. The intervention children reported significantly higher physical well-being (body well-being) compared to preterm controls. Likewise, the parents in the intervention group perceived their children as having significantly higher emotional well-being and a better school-related life compared to the preterm control group [28]. The hypothesis that MITP-M may generate a better quality of life and have positive long-lasting effects among preterm born children was supported. 

### 4.4. Stress in the Family

Stress in the parent-child relationship was measured by the *Parenting Stress Index* (PSI) [29]. The instrument is standardized with parents and children who range in age from 1 month to 12 years of age in different cultures and the instrument is considered to be reliable across cultures. PSI is a self-report questionnaire consisting of 101 items scored on a five-point Likert scale. The PSI consists of a Child Domain and a Parent Domain. The Child Doman reflects parental stress related to the child’s individual characteristics such as distractibility/hyperactivity, demandingness and mood. The parent domain reflects parental stress in connection with the parental role such as competence, attachment, role restriction and depression. At year 1 corrected age, there was a significant reduction of stress reported by mothers and fathers in the intervention group, compared to the preterm control group [30]. In the intervention group, reported stress was at the same level as for the term control group. These results were confirmed at age 2 [6]. Both parents in the intervention group reported significantly lower stress than the control group. In another report from the same study following the children from 6 months to 9 years of age, both parents in the intervention group reported parenting stress at similar levels as the term control group on all follow-ups and the agreement between parents enhanced over age [31].

Reduced stress in the family is assumed to increase parents’ sense of well-being and improve their competence in understanding individual characteristic of their infants, especially differences in temperament. In an earlier study, it was reported that the mothers’ level of stress largely determined how they perceived their infants’ temperament (IBQ) at 6 and 12 months [32,33]. There were strong correlations between stress and negative reactivity only in the preterm control groups. In the intervention group, MITP-M seemed to sensitize mothers to the temperamental regulatory competence of their preterm infants. The study suggests that mothers can benefit from intervention programs with respect to competence in “reading” their infants [33]. It was also reported that intervention mothers had weaker stress-behavior associations than control mothers [34]. 

## 5. Discussion

As mentioned in the introduction, a meta-review of the effectiveness of various intervention programs for preterm infants indicated the MITP as having the second most frequent positive impact across outcomes [1]. TISP presents evidence from a larger sample and supports the finding that MITP-M is an effective intervention. The findings showed that there was no effect on cognitive functioning at 2 years of age, which seems reasonable in light of a transactional model of development. Good circles of interaction implemented in accordance with the intervention procedures may be assumed to work for some time before developmental outcomes are affected (“sleeper effects”). It is also well known that the BSID have low predictive validity and several studies have shown that, until 2 years of age, correlations between scores on the BSID and later IQ-scores are low (around 0.30) [35]. However, the program had an effect on cognitive outcome in mean IQ-scores at 3 and 5 years of age, and the difference was within the clinically significant range. At 7 years of age, the only significant difference was found on the Verbal Comprehension Index and at 9 years of age, and the differences between the groups were rather small. This was an unexpected finding, which deviates from the findings in the Vermont study where IQ-scores increased over age [2,3]. A closer look at the results in our study indicates that the small difference between the groups may be explained by lack of power in the analysis. However, it should be pointed out that this finding is in line with a meta-analysis, which did not find effects of intervention after 5 years of age [36]. Interestingly, school achievements at 9 years of age in our study were at statistically same level as for term controls. 

Another important finding was that MITP-M affected both mothers’ and fathers’ perception of their infants on several temperamental constructs, and parents in the intervention group reported lower levels of negative emotionality in their children than the control group. This aligns perfectly with the mechanisms behind the transactional model. Infants in the preterm intervention group also scored significantly higher on joint attention that again helps infants to create advantageous learning environments for themselves. At this point, the reciprocal impact between cognitive and social development is demonstrated.

It was found consistently lower scores on all syndrome scales (CBCL) in the intervention group compared to the control group at 2 years of age but none of the differences were significant. As already mentioned, non-optimal infant-parent interactions may possibly have to last for some time before behavioral problems are more clearly revealed. This interpretation is supported by the fact that at 5 years significantly more infants in the control group scored within the clinical area on both instruments (CBCL, SDQ). At 7 and 9 years of age, both parents and teachers in the intervention group reported difficulties on the same level as term controls. An interesting finding was that at this age level, the intervention group had a significantly better quality of life. These findings confirm the assumption that the MITP-M may have a positive long-term effect on reducing the risk for behavioral problems and the intervention seems to increase the quality of life for preterms. However, a recent study did not find clear long-term effects of the MITP on behavioral outcomes [37]. Given the importance of developing age appropriate behavioral skills, more research is needed to address the broader aspects of relational factors at home that create better conditions for desired behavioral outcomes.

Already at 6 months corrected age, both mothers and fathers in the intervention group reported a significant reduction of stress which was at the same level as for the term control group and this effect was maintained up to 9 years of age. This effect is plausible in light of the character of the intervention since the MITP-M focused on enabling the parents to interpret and act according to the cues of their infants. This interpretation is supported by the fact that the mothers’ level of stress determined how they perceived the temperament of their infants since there was a strong correlation between stress and negative reactivity only for the preterm control group. Probably, the procedure has strengthened the parent-infant relationship, making the parents feel more competent in handling their preterm infants. To our knowledge TISP was the first study which demonstrated a sustained effect of early intervention on parenting stress in low birth weight children. Following this, other studies have shown positive impacts of the original version of MITP on maternal outcome (e.g., stress, sensitivity and responsiveness) as well as infant outcomes (e.g., cognitive development) [1].

Even if effects of different versions of the MITP of have been demonstrated, it is not clear *which* elements in the program are most effective. In our modified version, it may be the initial debriefing session. In our and other studies, it may also be the hospital sessions, the home-visits or a combination of the strategies. This insecurity has implications for clinical practice as well as future research. 

Concerning clinical practice, one might be restrained to recommend the whole program due to the economic costs. Yet, concerning the positive and sustained effect the intervention had on stress in the family, it would be meaningful to implement at least a part of the program during the stay at the Neonatal Intensive Care Unit. Since the home-visits were the most expensive parts of the intervention, there may be a better opportunity for training of the parents in how to understand and interpret their infants’ individual characteristics and cues in light of a cost-benefit analysis. Referring to the earlier mentioned meta-review on the effectiveness of early intervention programs, it would be a good idea to combine sessions from different programs. Kangaroo Care, which is a method where the parents are holding the baby in an upright position against a parent’s bare chest, has shown to be the most powerful intervention across all outcomes [1]. This relatively simple, low-cost intervention could be combined with sessions from the MITP-M.

Considering the lack of evidence concerning which sessions in intervention programs are most effective, future research should, among other things, trial combinations of elements from various interventions. The heterogeneity in intervention as well as outcomes calls for constructing integrated programs for parents and their preterm infants.

### Strengths and Limitations

An obvious strength of TISP is that the infants were recruited from a defined geographical area. In fact, almost all preterm infants born in this area (96%) were eligible for inclusion making the study a population-based randomized, controlled trial with blinded assessors and high follow-up rates. In addition, both parents were involved in more sessions relative to comparable studies.

A possible weakness is that birth weight rather than gestational age was used as inclusion criterion. The main argument for this decision was that it would have taken a disproportionately long time to recruit the subject if birth weight had not been chosen. As a result, some more mature growth-restricted infants were included making the conclusion somewhat more difficult to generalize. It should be emphasized that the growth-restricted infants were evenly distributed between the two preterm groups. Accordingly, the recruitment procedure should not influence the group differences reported.

## 6. Conclusions

The MITP-M seems to be an effective intervention affecting cognitive as well as social development. The program prevents development of behavioral problems and increases quality of life for preterm children. An especially strong and sustained effect, lasting from the first year until 9 years of age, is seen on low levels of stress in intervention families, which is comparable to the stress level in families with full-term born infants. Neonatal care policy should recommend interventions which have shown clear positive effects on outcomes.

## Abbreviations

BSID	Bayley Scales of Infant Development
CBCL	Child Behavior Checklist
CCTI	Colorado Childhood Temperament Inventory questionnaire
CRPP	Child Rearing Practice Report
EAS	Emotionality, Activity and Sociability questionnaire
ESCS	Early Social Communication Scales
IBQ	Infant Behavior Questionnaire
PSI	Parenting Stress Index
KINDL	Questionnaire for measuring quality of life for children and adolescents
MITP	Mother-Infant Transaction Program
MITP	M Mother-Infant Transaction Program Modified
SDQ	Strengths and Difficulties Questionnaire
TISP	Tromsø Intervention Study on Preterms
TRF	Teacher Report Form
WIPPSI-R	Wechsler Preschool Primary Scale of Intelligence
WISC-III	Wechsler Primary Intelligence Scale for Children
